# The effect of weather on the decision to migrate from stopover sites by autumn-migrating ducks

**DOI:** 10.1186/s40462-018-0141-5

**Published:** 2018-11-27

**Authors:** Benjamin J. O’Neal, Joshua D. Stafford, Ronald P. Larkin, Eric S. Michel

**Affiliations:** 1 Illinois Natural History Survey, Prairie Research Institute, University of Illinois, Champaign, IL USA; 2 Frank C. Bellrose Waterfowl Research Center, Illinois Natural History Survey, Institute of Natural Resource Sustainability, University of Illinois, Havana, IL USA; 3 Department of Natural Resource Management, South Dakota State University, Brookings, SD USA; 4 Present address: Franklin College, Franklin, IN USA; 5 Present address: U.S. Geological Survey, South Dakota Cooperative Fish & Wildlife Research Unit, Department of Natural Resource Management, South Dakota State University, Brookings, SD USA

**Keywords:** *Anatidae*, Migration, Radar, Remote sensing

## Abstract

**Background:**

Previous investigations of autumn-migrating ducks have reported weak connections between weather conditions and the decision to migrate from stopover sites. We leveraged relatively new weather surveillance radar technology to remotely detect departures of discrete groups of various species of migratory dabbling ducks (*Anatidae*) in autumn to more directly assess the effect of specific weather conditions on departure from discrete stopover sites.

**Methods:**

Using radar data collected over fifteen years (1995–2009), we documented a consistent phenomenon where a single, identifiable group departed from our study area on 30% of days during the autumn study period, and no ducks departed on the other days. We gathered weather variables from nearby stations and used them to develop competing models to explain temporal patterns of departure versus non-departure to better understand the potential mechanisms associated with binomial patterns of departures.

**Results:**

The best approximating model of departure probability was our integrated model, which included variables accounting for wind aloft direction favorable for departure (i.e., tailwind), absence of precipitation, and a partially or completely clear sky. The integrated model accounted for all model weight in the candidate set and explained 55% of the variation in departure probability. Estimated probability of departure was 0.76 after parameterizing the best model with favorable conditions for all covariates.

**Conclusions:**

Our results contrasted those of previous studies of autumn duck migration as a small set of simplistic, extrinsic conditions substantially influenced departure decision.

**Electronic supplementary material:**

The online version of this article (10.1186/s40462-018-0141-5) contains supplementary material, which is available to authorized users.

## Background

Throughout the course of migration, birds, bats, and other organisms make many important decisions, including where to go, how long to stay, and when to leave. Each of these decisions affects the others and ultimately contributes to the fitness of the individual [1–5]. The decision of when to migrate is especially important because it requires forfeiting existing conditions, enduring conditions aloft, and assuming risk regarding the conditions of an unseen destination. Because the timing of departures during spring has a direct effect on reproductive output, many bird species have developed endogenous mechanisms to control the timing of migration toward the breeding grounds [6]. During autumn, however, birds seem to rely more on external factors, such as food availability, predation risk, social context, and weather [7]. We contend that examining the relationships between these external factors and migratory responses can provide insight into which factors are important to specific taxa at which times and in which places [8, 9].

Among the environmental factors that affect the timing of autumn migration, weather has been identified as a key factor for many avian taxa [10, 11]. Despite the extensive study of migration and weather [12], the specific role of weather in the regulation of autumn migration remains uncertain for some major avian taxa including waterfowl guilds such as dabbling ducks (but see [13, 14]).

One reason for our limited understanding of the influence of weather on duck migration is the fact that the timing of departure in ducks is especially complicated. Unlike passerines, which often operate under a time-minimization strategy [15], autumn-migrating ducks typically remain at mid-migration stopovers for multiple days, even though weather conditions suitable for migration appear present [16, 17]. The timing of duck migration is further complicated by hunting pressure [18] and habitat quality [19].

The few published studies of migrating ducks suggest that weather has a relatively minor effect on the timing of duck departure. Indeed, Beason [20] asserted that weather “plays only a minor role in influencing autumn migration”, and Bellrose [14] argued that “factors other than weather were responsible for initiating most departures of ducks.” Nevertheless, weather has been shown to influence the distributions of ducks during autumn and winter [21–23], the timing of departure in other avian taxa [11, 12], and the overall migratory phenology of birds in general [24]. It follows that weather would also play a part in the temporal variation in the departure of ducks.

Previous studies have likely failed to identify the effect of weather because of the methods used to quantify the timing of departure. Rather than examining the variation in migration at a specific place and time each day, Beason [20] combined data from six radars spanning much of the southwestern United States and analyzed the peak migration traffic rate for each night across the whole study region. In addition, the birds in Beason’s [20] study were only identified generally as “non-passerines.” Bellrose [14] attempted to infer the daily magnitude of migration based on weekly changes in abundances of ducks, which almost certainly changed between surveys based on our understanding of turnover rates. He also tried to quantify the magnitude of the migratory response by analyzing the size of each daily departure relative to the total number of migrants for each year [14]. This approach implies that the magnitude of a migratory response at one time of year is relative to the amount of migration occurring at another time of the year, which violates the assumptions of independence for the linear regression used to analyze his continuous dependent variable. In this study, we explored how a dependent variable describing the day-to-day presence or absence of a duck departure event from an explicit location could substantially improve upon previous studies. Based on this improved approach, we predicted that there would be a measurable relationship between local weather conditions and the day-to-day timing of migration in autumn-migrating ducks. We used weather surveillance radar (WSR) to monitor the egress of ducks from a specific midcontinent stopover site over multiple years during autumn, and we relate the timing of these departures and non-departures to relevant weather conditions.

Tailwind was documented as the primary weather condition affecting the propensity for migration in many bird species, including ducks, due to its substantial effect on the net energetic cost of migration [11, 20, 25, 26]. A recent study has shown a mechanistic connection between birds’ perceived degree of wind assistance, their baseline corticosterone levels, and their associated departure probabilities [27]. With respect to the direction of the wind, Erni et al. [28] reported birds distinguished between favorable and unfavorable wind conditions rather than graded wind on a continuous scale of favorability. This suggests that ducks stopping over at our study area might well be influenced by a simplistic binary mechanism associated with the direction of winds aloft relative to their preferred direction of departure.

Precipitation is another factor with the potential to regulate avian migration probability due to its impedance of flight, influence on thermoregulation [11, 15, 16], and potential to cause mortality [29]. Therefore, we hypothesized that departure decisions among autumn-migrating ducks might be influenced in a direct way by the simple presence or absence of precipitation [30].

Departure probability can also be influenced by weather conditions that affect the orientation mechanism. For example, stars are thought to aid in the orientation of ducks and other birds during flight, so their obstruction by clouds could reduce the probability of departure [31, 32]. Therefore, we hypothesized that duck departure might be influenced by the amount of cloud clover as well as the simple ability or inability to see some portion of the night sky and its visual cues. In addition to upward visibility, departure can also be influenced by migrant birds’ ability to see terrestrial orientation. This ability is at least partially determined by the height of the cloud ceiling. Given the fact that ducks migrate through our study area at 490 ± 163 m [33], we hypothesized that cloud ceilings below a threshold of 600 m might inhibit departure in a categorical fashion.

Air temperature is another important factor that affects the energetic balance of birds [34, 35], the progression of duck migration, and the latitudinal distribution of migrants throughout a season [21, 23]. However, the role of temperature as a proximate cue for migration initiation on a fine temporal scale (i.e., daily) is uncertain, so we included this factor in our investigation [14]. Additionally, the difference in temperature from the preceding day is another potential cue for duck departures. Some migrants, especially the early-season obligate migrants in our study, may have an environmental temperature at which they are no longer comfortable remaining at a stopover. Therefore, the minimum temperature for a given day may also influence the probability of departure in our study system. Barometric pressure, as well as the change in pressure, may also serve as proximate cues for future conditions at current and future locations of birds [12].

In addition to weather factors, there are also habitat factors that have been shown to influence duck migration [36]. Production of plant foods for waterfowl in our study system can vary considerably from year to year based, on a highly dynamic hydrology regime. An index for the quantification of this annual variation in foraging habitat quality has been developed and validated within our study system [37]. Concurrent studies at our study site have shown a strong relationship between this foraging habitat index and dabbling duck stopover duration [36]. If the duration of stay is influenced by this factor, it is certainly possible that the probability of departure is as well [36].

In addition to the simplistic mechanisms above, some external factors may also work together in an additive fashion to influence stopover. For example, stopover habitat suitable for migratory ducks is often isolated spatially [38]. The ability to orient to suitable habitat may be dependent on the ability to observe multiple orientation cues in the sky and/or on the ground. If this is the case, we might expect to see the height of the cloud ceiling (relative to ducks’ preferred heights (400–600 m; [12]), along with amount of cloud cover and its influence on sky visibility, combining to influence the probability of migratory departure [20]. Departure probability can also be influenced by a combination of weather conditions related to the thermal environment of a bird. Current temperature and wind speed, along with anticipated future temperature (as indicated by changes in temperature from the preceding day), can have substantial effects on thermoregulation in ducks during autumn and thereby have a substantial effect on the decision to depart from a stopover [14, 20]. Finally, it is possible that factors related to energetic efficiency of flight as well as the capacity for effective orientation might influence departure probability in an additive fashion.

Our specific objectives were to: 1) screen WSR data from central Illinois to identify duck departures from a major stopover along the Illinois River; 2) develop competing models to evaluate relationships between the timing of departures and weather; 3) parameterize the relationships between individual covariates and departure probability to understand the magnitude of the effects of particular variables.

## Methods

### Study site

**Fig. 1:**
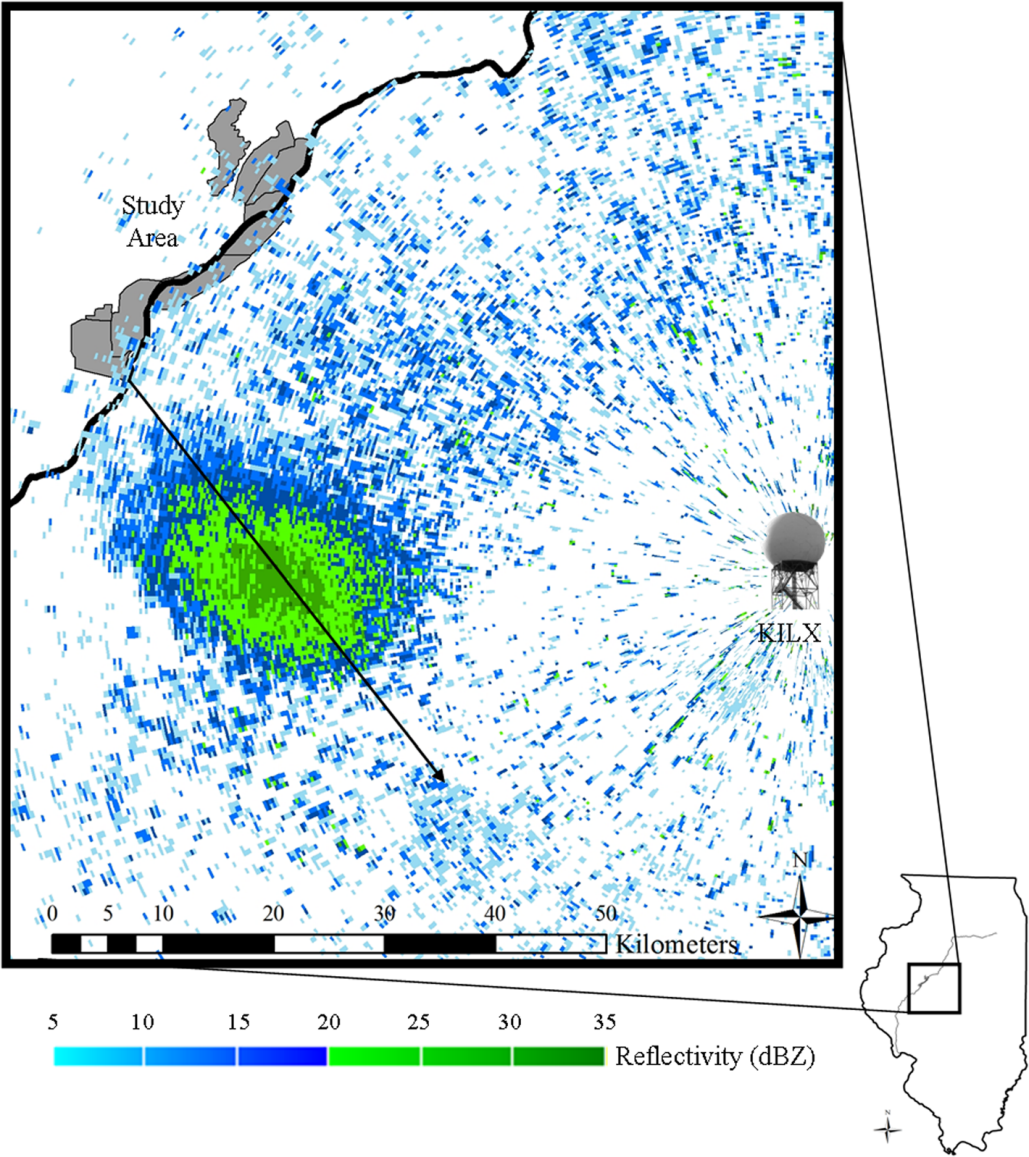
Weather surveillance radar in Lincoln, Illinois (KILX) reveals a representative departure of approximately 20,000 ducks from our Illinois River study area, Illinois, USA, at 2340, 08 November 2008 (0.5° elevation reflectivity scan). The identity of these aerofauna was accomplished through targeted ground-truthing using aerial inventories of the stopover site and thermal infrared and portable radar observations along the flight path (O’Neal et al. 2010). Black arrow indicates departure track (150°)

We monitored dabbling duck departures from a 14,431-ha complex of wetlands and backwater lakes in central Illinois (40.376055°N, − 90.027238°W; Fig. 1). Our study area contained several wetland types, including areas managed for growth of moist-soil plants [39], large areas of open water with submerged aquatic vegetation, floodplain forests, and shallow-water lakes [37]. Over the last several decades, Chautauqua NWR has been the most important waterfowl refuge in Illinois with respect to use, and has been designated a Globally Significant Bird Area [37, 38]. In 2006, The Nature Conservancy and the U.S. Fish and Wildlife Service restored an additional 4000 ha (Emiquon Preserve, Wilder Tract, Thompson Lake, Flag Lake), substantially increasing the amount of habitat for migratory waterfowl within this complex. These key stopovers and the surrounding wetlands are part of a migratory flyway that is vital to numerous species of birds and representative of other major waterfowl flyways across North America. Among the duck species that utilize this area, dabbling ducks (Tribe Anatini) accounted for 90% of waterfowl use during autumn migration periods from 1995 to 2008, according to aerial inventories [40]. The complex was 60 km west of a WSR unit (KILX) located in Lincoln, Illinois.

### Quantification of departure

We downloaded level II data recorded by the KILX unit during autumn 1995–2009 from the National Environmental Satellite, Data, and Information Service (https://www.ncdc.noaa.gov/nexradinv/). We screened all reflectivity scans (24 h/day, ≥144 scans/day) from October 1–December 31, 1996–1998, 2003, and 2006–2009, identifying duck departures according to established ground-truthed criteria [36, 41]. We could identify targets to tribe (dabbling ducks), but not to species. Although migratory behaviors may differ among dabbling duck species [31], their overall responses to weather are likely similar [23, 42]; therefore, we conducted our study at the taxonomic resolution of tribe. All departure events detected in the first eight years that we analyzed appeared on radar shortly after sunset (mean ± SD = 44 ± 6 min). Based on this finding and previous studies showing that the vast majority of dabbling duck migrations occur shortly after sunset [20, 25], we analyzed departures from an additional seven years (1995, 1999–2002, and 2004–2005) based solely on a two-hour period surrounding sunset (1700–1900 CST/1800–2000 CDT/2300–0100 GMT; ~ 12 scans/day). Because our identification of radar targets partially relied on species composition identified by aerial inventories, we analyzed migratory events from the mean date at which aerial inventories were initiated each year (15 October ±2 days [SD]) through the date on which our study area was estimated to have frozen over based on field observations and daily surface temperature (A. Yetter, Illinois Natural History Survey, unpublished data). The median date of the final departure event observed on radar from 1995 to 2009 was December 5.

### Weather data

The National Oceanic and Atmospheric Administration (NOAA) collects and archives a set of standard weather variables intended for various uses. Many of the variables that are collected have relevance for the study of migration based on existing literature. Among these variables, we identified a sub-set of variables that contained adequate temporal coverage to support statistical analysis. Hourly weather variables (precipitation, cloud cover, temperature, pressure, cloud height, and surface wind) were collected in Peoria, Illinois, about 40 km N of our study site (Table 1 and Additional file 1). Because departures occurred at similar times within and among years (2300–0000 GMT; [41; Additional file 1]), we analyzed hourly weather data for the same times on both departure and non-departure days (~ 1700 CST/1800 CDT/2300 GMT). Winds aloft variables were gathered from the nearest radiosonde station, which was located in Lincoln, Illinois, about 60 km east of our study area (Table 1 and Additional file 1). These data were collected at 0000 GMT (1800 CST/1900 CDT) the night of each departure. We obtained data on mean daily temperature from Havana, Illinois (about 10 km SE of study area; Table 1 and Additional file 1).

**Table 1:** Definition of variables used to explain the probability from stopover sites by autumn-migrating dabbling ducks from 1995 to 2009

Variable Name	Variable Definition
Winds aloft index	Binary description of the direction of winds aloft at an elevation within the known cruising altitude of ducks in this region (433 m above ground level [26]). Favorable winds (1) are those that are following relative to the preferred direction of departure from study site 151.8° ± 0.71° (mean ± se), and unfavorable winds (0) are those that are opposing.
Precipitation index	Binary description of precipitation status with 1 = No precipitation observed at time of departure (2300 GMT), 0 = Precipitation observed at time of departure
Cloud cover	Proportion of the sky covered by clouds at time of departure (0/8–8/8)
Cloud cover index	Binary index describing the amount of cloud cover. When stars were completely obscured (8/8 cloud cover), an index of 0 was assigned; If at least some portion of the stars were visible (0/8–7/8 cloud cover), an index of 1 was assigned
Cloud height index	Binary index of cloud ceiling height with 1 = Cloud ceiling> 600 m AGL and 0 = Cloud ceiling< 600 m
Temperature	Surface air temperature at time of departure (°C)
Difference in daily mean temperature	Difference in mean daily temperatures (°C) between the 24-h period immediately preceding a departure/non-departure event and the 24-h period prior to that day
Minimum daily temperature	Minimum daily temperature (°C)
Barometric pressure at dusk	Barometric pressure at dusk (mb)
Change in barometric pressure	Change in barometric pressure from 6 h prior to departure (1700 GMT) to time of departure (2300 GMT)
Foraging habitat index	Qualitative index of waterfowl food production measured within study area in August/September with 0–2 = no or poor food production, 3–4 = fair, 5–6 = good, 7–8 = very good, and 9–10 = excellent [NA = Not available]
Surface wind speed	The rate of horizontal travel of air past a fixed point (m/s)
Julian date	Julian date

### Data excluded from analysis

Our study radar (KILX) did not collect data on 11 possible nights and was obstructed by weather [42] on an additional 11 nights. Weather data were missing for 107 days. Our modeling analysis prevented use of days lacking data for any covariate, so these days were excluded entirely from analysis, resulting in a final sample of 723 out of 852 possible days (Additional file 1). Omitted dates were distributed within and among years in a non-systematic manner, and we do not believe their omission biased the analysis of departure probability [43].

### Model development

Using the weather variables described above (Table 1) [11, 12], we developed a set of a priori candidate models to explain the timing of dabbling duck departures (Table 2). Our candidate model set included one univariate model for each fixed predictor as well as 3 multivariate models. Our response variable had a binomial distribution in which nights with departures were assigned a value of 1, and those without were assigned a value of 0 [44]. Although the timing of migration is generally controlled less by photoperiod in the autumn than in the spring [45], some studies have shown an effect of seasonality on daily departure probability [20]; therefore, we included year as a random variable within the logistic framework to account for annual variation.

**Table 2:** Names of hypotheses and variables included for each hypothesis used to explain the probability from stopover sites by autumn-migrating dabbling ducks from 1995 to 2009. Predicted direction of effect included in parentheses

Hypothesis Name	Variables Included
WINDSALOFTINDEX	Winds aloft index (+)
PRECIPINDEX	Precipitation index (−)
CLOUDCOVER	Cloud cover (−)
CLOUDCOVERINDEX	Cloud cover index (+)
CLOUDHEIGHTINDEX	Cloud height index (+)
TEMPERATURE	Temperature (−)
MEANDAILYTEMPCHG24HR	Difference in daily mean temperature (−)
DAILYMINT	Minimum daily temperature (−)
PRESSURE	Barometric pressure at dusk (+)
PRESSURECHANGE	Change in barometric pressure (+)
HABITAT	Foraging habitat index (−)
ORIENTATION	Cloud height index (+), Cloud cover (−)
THERMAL	Temperature (−), Difference in daily mean temperature (−), Surface wind speed (+)
INTEGRATED	Winds aloft index (+), Precipitation index (−), Cloud cover index (+)
DATE	Julian date (+)
Null	Intercept only

### Statistical analyses

We modeled dabbling duck departure using a logistic regression within a mixed model framework via the glmer function in the lme4 package in Program R [46, 47]. We also fit models with a binomial distribution and logit link function. We examined covariates for multi-collinearity based on variance inflation factors (VIF) using the vif function in the car package in Program R [48], but none were > 1.20, so we retained them all [49]. We evaluated candidate models using Akaike’s Information Criterion (AIC) to determine best approximating and competing models [50]. We considered models within Δ 2 AIC units as competing [50]. We derived AIC values, AIC weights (*w*_*i*_), number of parameters, and model weights in the MuMIn package in Program R. We evaluated model fit by computing marginal (fixed effects only) and conditional (fixed and random effects) *R*^*2*^ values according to Nakagawa and Schielzeth [51] using the r.squarredGLMM in the MuMIn package. We calculated odds ratios (OR) and their 95% confidence intervals (estimated using the Wald method) for covariates in the best model to evaluate their influence on migratory departure (i.e., the odds of departure occurring relative to the odds of non-departure).

## Results

Over our 15-year study period, ducks departed on an average of 30% of nights each year, with a total of 216 departures out of all 723 nights. The “integrated” model best described the timing of departure relative to weather, capturing all of the model weight (*w*_*i*_ = 1.00) and explaining more than half of the daily variation (Conditional *R*^2^ = 0.55). All other models were ≥ 39.1 ΔAIC (Table 3). Wind aloft (β = 3.560, 95% CI = 3.012–4.173), cloud cover (β = 1.016, 95% CI = 0.591–1.451), and precipitation (β = 2.581, 95% CI = 0.954–5.487) positively affected departure.

**Table 3:** Candidate weather models formulated to explain variation in the timing of departure among autumn-migrating dabbling ducks in Illinois River valley, as detected by weather surveillance radar, 1995–2009, ranked by ascending Akaike’s Information Criterion (AIC). We included YEAR as a random effect in each model. *w*_*i*_ indicates model weight and *K* indicates number of parameters used in each model

Model	ΔAIC	*w*_*i*_	*K*
WINDALOFTINDEX + CLOUDCOVERINDEX + PRECIPINDEX	0.00	1.00	3
WINDALOFTINDEX	39.10	0.00	1
PRESSURECHANGE	182.73	0.00	1
HABITAT	233.23	0.00	1
TEMPERATURE + MEANDAILYTEMPCHG24HR + SURFWINDSPD	235.84	0.00	3
MEANDAILYTEMPCHG24HR	241.78	0.00	1
TEMPERATURE	267.19	0.00	1
PRECIPINDEX	270.24	0.00	1
PRESSURE	272.39	0.00	1
CLOUDHEIGHTINDEX + CLOUDCOVER	275.27	0.00	2
CLOUDHEIGHTINDEX	276.57	0.00	1
CLOUDCOVER	285.39	0.00	1
CLOUDCOVERINDEX	285.66	0.00	1
DATE	287.32	0.00	1
DAILYMINT	292.71	0.00	1
Null	294.81	0.00	0

Based on coefficient and odds ratio estimates for the best model, ducks were more likely to depart with following winds, no precipitation, and less cloud cover (Table 4). The winds aloft index covariate had the highest OR, indicating the odds of ducks departing with a flight index of 1 as opposed to not migrating were 35.2 to 1 (95% CI 20.0–62.6). Holding all covariates constant, the probability of ducks departing assuming a flight index of 1 (all conditions favorable) was 0.76.

**Table 4:** Estimated coefficients and 95% CIs as well as odds ratios and 95% CIs for covariates of the best model (INTEGRATED) explaining variation in daily departure probability of autumn-migrating dabbling ducks in Illinois River valley, as detected by weather surveillance radar, 1995–2009

Variable	Coefficient	95% CI	Odds ratio (OR)	95% CI for OR
WINDALOFTINDEX^a^	3.56	2.98–4.14	35.2	19.7–62.6
CLOUDCOVERINDEX^b^	1.02	0.59–1.45	2.8	1.8–4.2
PRECIPINDEX^c^	2.58	0.54–4.63	13.2	1.7–102.2

^a^wind aloft index (following winds yielded a value of 1, opposing winds yielded a value of 0)

^b^cloud cover index (complete overcast was coded as 0, and partially clear skies as 1)

^c^precip index (absence of rain at time of departure was coded as 1, and presence of rain as 0)

## Discussion

Our integrated model, which included following winds aloft, no precipitation, and a less than complete cloud cover, was clearly superior among candidate models describing migratory departure in autumn-migrating dabbling ducks. This model outperformed simpler models in spite of being more parameterized. It also explained over half of the variation in daily departure timing versus non-departure, which was considerably more than previous studies that sought to quantify the relative influence of extrinsic weather conditions on departure decisions of ducks ([14]: *R*^2^ = 0.19; [17 (20)]: *R*^2^ = 0.10). Contrary to the results of most previous studies on waterfowl, our results suggested weather was indeed an important factor in the timing of autumn migration of dabbling ducks at our study site.

**Fig. 2:**
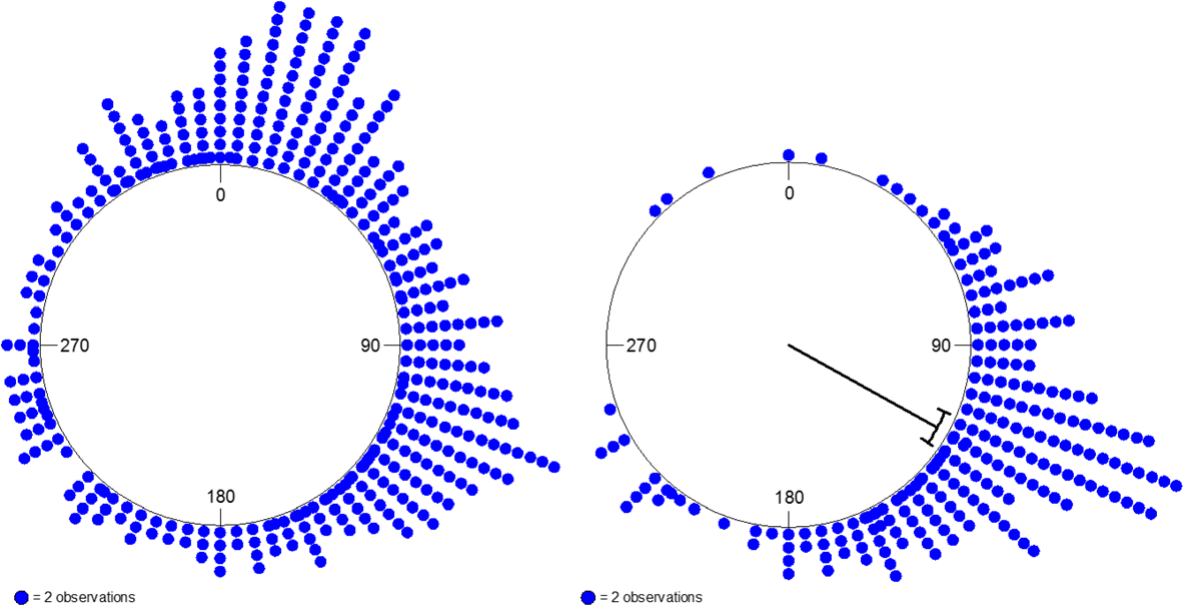
Frequency distribution of the direction that the winds aloft (433 m Above Ground Level) are blowing toward at 0000 GMT on each night included in our analysis (left) compared to the frequency distribution of the direction that winds aloft are blowing toward at 2400 GMT on a sub-set of nights on which we observed a departure event

The best model was dominated by the winds aloft index covariate, which had the highest OR of all the covariates. The following or opposing nature of the wind aloft alone explained nearly half of the variation in departure timing (*R*^2^ = 0.47; Fig. 2). The use of wind data from the altitudes at which ducks migrate rather than the surface likely improved the fit of this model compared to earlier studies [14, 52]. Precipitation also had an important effect, as indicated by the magnitude of the OR. The large CI about the OR of precipitation elucidates the biological nature of its effect. Namely, the presence of precipitation consistently halted migration, but its absence was not a good predictor for the occurrence of migratory departure. Although migratory behaviors differ among Anseriformes and Passeriformes, the weather variables that determine the timing of autumn migration appear to be similar in both taxa [53].

Our results suggest that conditions associated with flight may, in some contexts, be equal to or more important than local habitat conditions at a stopover. This is in contrast to typical thinking regarding duck migration in the mid-continent [14, 37, 38], which has typically emphasized the importance of site-specific conditions that influence birds’ access to forage (e.g., snow cover and ice) [20, 23]. The general unimportance of weather severity at the local stopover versus the suitability of flight conditions aloft in our study, was probably due in part to the inclusion of early-migrating species (i.e., northern pintail [*Anas acuta*], green-winged teal [*A. crecca*], American wigeon [*A. americana*], gadwall [*A. strepera*], and northern shoveler [*A. clypeata*]), as opposed to only mallards (*A. platyrhynchos*). The presence of substantial emergent marsh habitat within the Emiquon Preserve is largely responsible for the abundance of non-mallard dabbling ducks within our study complex [54]. Mallards often depart only when forced to do so by severe weather that degrades site-level conditions. However, the suite of dabbling duck species we examined included many obligate migrant species, which would have been more likely to move through the region prior to onset of inhospitable site conditions (i.e., freezing temperatures, frozen surface water, and snow cover). Therefore, the taxonomic composition of our study subjects may partly explain why flight condition models explained departure better than site condition models. The fact that we had to collectively evaluate both early- and late-migrating species of dabbling duck likely explains why DATE did not perform well as an explanatory model for the timing of departure in the entire guild.

Although 55 % of the variation in the timing of departure was explained by our best weather-based model, 45 % was unaccounted for. Weather conditions such as wind direction and precipitation can vary over relatively small spatial scales. As such, there was likely error associated with the spatial disconnect between the stopover site of interest and the site at which weather data were collected. There may have been additional unexplained variation associated with a “contrast effect,” [8, 55, 56] which results in higher departure probabilities when favorable conditions follow unfavorable conditions (i.e., high contrast) rather than similar favorable ones (i.e., low contrast). We did not attempt to model this effect due to its high correlation with weather variables [11]. Disturbance caused by interactions with hunters may also have contributed to unexplained variation in departure timing [15, 18, 44, 57]. Finally, food availability as determined by forage production and density-dependent competition likely affects the overall amount of time ducks spend at a stopover, and therefore may have contributed to variation in departure timing in our study [36].

## Conclusions

Our results address some questions regarding the importance of weather conditions in the departure decisions of ducks, but they also raise several others. For example, our results indicated direction of winds aloft was a key factor in departure decisions, but it is unknown how ducks might perceive or sample wind conditions aloft prior to departure. Additionally, the role of non-weather factors (e.g., body condition and hunting) in departure decisions remains unclear and warrants further attention. Finally, our research was conducted during autumn, but considerable questions remain regarding spring migration in ducks [58].

## Additional file

Additional file 1: Migratory departure status (0 = no departure, 1 = departure) for each day included in our analysis and the associated weather conditions for those days (WINDALOFTDIRFROM: Direction from which wind is flowing at 433 m above ground level; WINDALOFTINDEX: 1 = Wind aloft is following relative to preferred direction of travel, given observed departure tracks over entire study period, 0 = Wind aloft is opposing relative to preferred direction of travel, given observed departure tracks over entire study period; PRECIPINDEX: 1 = No precipitation observed at time of departure [2300 GMT], 0 = Precipitation observed at time of departure; CLOUDCOVER: number of eighths of the sky covered by clouds at time of departure; CLOUDCOVERINDEX: 1 = Between 0/8 and 7/8 of the sky is covered (i.e., stars are partially visible), 0 = 8/8 of the sky is covered (i.e., complete overcast); TEMPERATURE: Surface air temperature at time of departure (°C); TEMPCHANGE: Difference in mean daily temperature (°C) between the 24-h period immediately preceding a departure/non-departure event and the 24-h period prior to that day; DAILYMINT: Minimum daily surface air temperature observed for the calendar day (°C); PRESSURE: Barometric pressure (mb) observed at the time of departure; PRESSURECHANGE: Change in barometric pressure over the six hours preceding typical departure (1700–2300 GMT); CLOUDHEIGHTINDEX: 1 = Cloud ceiling> 600 m, 0 = Cloud ceiling< 600 m; SURFWINDSPD: The rate of horizontal travel of air past a fixed point (m/s); HABITAT: Qualitative index of waterfowl food production measured within study area in August/September with 0–2 = no or poor food production, 3–4 = fair, 5–6 = good, 7–8 = very good, and 9–10 = excellent [NA = Not available]. (CSV 39 kb)
